# *catena*-Poly[[[bis­[4-(1*H*-1,3,7,8-tetra­azacyclo­penta­[*l*]phenanthren-2-yl)phenol-κ^2^ *N* ^7^,*N* ^8^]manganese(II)]-μ-naphthalene-1,4-dicarboxyl­ato-κ^2^ *O* ^1^:*O* ^4^] naphthalene-1,4-dicarboxylic acid hemisolvate monohydrate]

**DOI:** 10.1107/S160053680801074X

**Published:** 2008-04-23

**Authors:** Heng-Da Li, Xiu-Ying Li, Mao-Liang Xu, Seik Weng Ng

**Affiliations:** a Department of Chemistry, Jilin Normal University, Siping 136000, People’s Republic of China; b Xi’an Modern Chemistry Research Institute, Xi’an 710065, People’s Republic of China; c Department of Chemistry, University of Malaya, 50603 Kuala Lumpur, Malaysia

## Abstract

The 1,4-dicarboxyl­ate dianions in the title compound, [Mn(C_12_H_6_O_4_)(C_19_H_12_N_4_O)_2_]·0.5C_12_H_8_O_4_·H_2_O, bond to two 4-(1*H*-1,3,7,8-tetra­azacyclo­penta­[*l*]phenanthren-2-yl)phenol-chelated Mn atoms to form a chain that features the metal atom in an octa­hedral coordination geometry. Adjacent chains inter­act with the uncoordinated water mol­ecules to form a three-dimensional network. The naphthalene-1,4-dicarboxylic acid solvent mol­ecule, which is disordered about a centre of inversion, occupies the space within the network but is not bonded to the network. One NH group is disordered equally over two positions.

## Related literature

There are several studies of (2-phenyl-1*H*-1,3,7,8,-tetra­azacyclo­penta­[*l*]phenanthrene-chelated manganese dicarb­oxyl­ates (see, for example, Li *et al.*, 2008 ▶). The 4-hydr­oxy-substituted *N*-heterocycle forms an adduct with mangan­ese(II) terephthalate (see Che *et al.*, 2006 ▶).
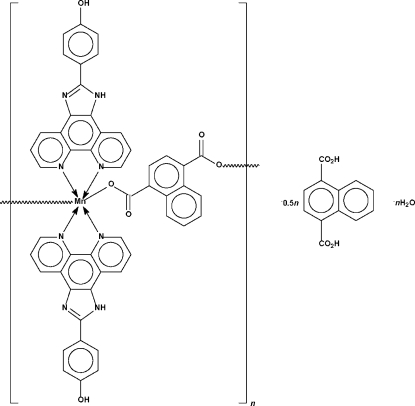


## Experimental

### 

#### Crystal data

[Mn(C_12_H_6_O_4_)(C_19_H_12_N_4_O)_2_]·0.5C_12_H_8_O_4_·H_2_O
*M* *_r_* = 1019.87
Monoclinic, 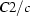

*a* = 48.398 (15) Å
*b* = 9.089 (2) Å
*c* = 20.598 (6) Å
β = 103.20 (1)°
*V* = 8821 (4) Å^3^
*Z* = 8
Mo *K*α radiation
μ = 0.38 mm^−1^
*T* = 295 (2) K
0.31 × 0.25 × 0.18 mm

#### Data collection

Rigaku R-AXIS RAPID diffractometer
Absorption correction: multi-scan (*ABSCOR*; Higashi, 1995 ▶) *T*_min_ = 0.820, *T*_max_ = 1.000 (expected range = 0.767–0.935)
40785 measured reflections
10014 independent reflections
5868 reflections with *I* > 2σ(*I*)
*R*_int_ = 0.084

#### Refinement

*R*[*F* ^2^ > 2σ(*F* ^2^)] = 0.060
*wR*(*F* ^2^) = 0.170
*S* = 1.03
10014 reflections
717 parameters
116 restraints
H-atom parameters constrained
Δρ_max_ = 1.35 e Å^−3^
Δρ_min_ = −0.42 e Å^−3^

### 

Data collection: *RAPID-AUTO* (Rigaku, 1998 ▶); cell refinement: *RAPID-AUTO*; data reduction: *CrystalStructure* (Rigaku/MSC, 2002 ▶); program(s) used to solve structure: *SHELXS97* (Sheldrick, 2008 ▶); program(s) used to refine structure: *SHELXL97* (Sheldrick, 2008 ▶); molecular graphics: *X-SEED* (Barbour, 2001 ▶) and *OLEX* (Dolomanov *et al.*, 2003 ▶); software used to prepare material for publication: *publCIF* (Westrip, 2008 ▶).

## Supplementary Material

Crystal structure: contains datablocks global, I. DOI: 10.1107/S160053680801074X/bx2132sup1.cif

Structure factors: contains datablocks I. DOI: 10.1107/S160053680801074X/bx2132Isup2.hkl

Additional supplementary materials:  crystallographic information; 3D view; checkCIF report

## Figures and Tables

**Table:** 

Mn1—O1	2.146 (2)
Mn1—O3^i^	2.108 (2)
Mn1—N1	2.282 (3)
Mn1—N4	2.245 (3)
Mn1—N5	2.265 (3)
Mn1—N8	2.307 (3)

**Table:** 

O1—Mn1—O3^i^	95.0 (1)
O1—Mn1—N1	87.1 (1)
O1—Mn1—N4	97.6 (1)
O1—Mn1—N5	97.9 (1)
O1—Mn1—N8	169.9 (1)
O3^i^—Mn1—N1	165.2 (1)
O3^i^—Mn1—N4	91.8 (1)
O3^i^—Mn1—N5	104.8 (1)
O3^i^—Mn1—N8	87.1 (1)
N1—Mn1—N4	73.4 (1)
N1—Mn1—N5	89.5 (1)
N1—Mn1—N8	93.4 (1)
N4—Mn1—N5	156.2 (1)
N4—Mn1—N8	92.2 (1)
N5—Mn1—N8	72.0 (1)

Symmetry code: (i) 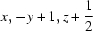.

**Table 2:** Hydrogen-bond geometry (Å, °)

*D*—H⋯*A*	*D*—H	H⋯*A*	*D*⋯*A*	*D*—H⋯*A*
O5—H5*O*⋯N2^ii^	0.82	1.93	2.737 (4)	168
O6—H6*O*⋯O1*W*^iii^	0.82	1.85	2.656 (4)	168
N3—H3*N*⋯O2^iv^	0.86	1.97	2.813 (4)	166
N6—H6*N*⋯O9	0.86	2.00	2.728 (6)	142
N7—H7*N*⋯O10^v^	0.86	1.83	2.685 (6)	178
O1*W*—H1*W*2⋯O1	0.82	1.94	2.754 (4)	173
O1*W*—H1*W*1⋯O4^i^	0.82	2.19	3.007 (6)	173

Symmetry codes: (i) 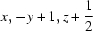; (ii) 
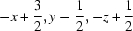
; (iii) 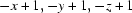; (iv) 
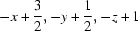
; (v) 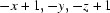.

**Fig. 1.**
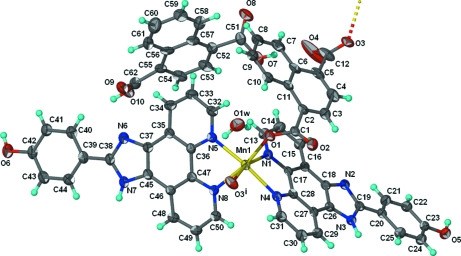
Thermal ellipsoid plot of Mn(C19H12N4O)2(C12H6O4).(C12H8O4)0.5.H2O; displacement ellipsoids are drawn at the 50% probability level, and H atoms as spheres of arbitrary radius. The naphthalene-1,4-dicarboxylic acid is disordered about a center-of-inversion. [Symmetry code i: x, 1 - y, 1/2 + z].

**Fig. 2.**
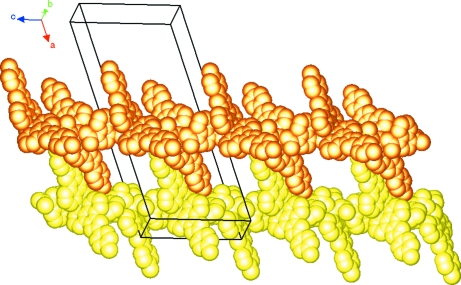
Chain structure of the manganese-naphthalene-1,4-dicarboxylate network as illustrated by OLEX (Dolomanov et al., 2003).

## supplementary crystallographic information

### Comment

There are manganese dicarboxylate adducts of complexes of 2-phenyl-1*H*-1,3,7,8-tetraazacyclopenta[*l*]phenanthrene (Li *et al.*, 2008). The 4-hydroxy substituted ligand forms an adduct with manganese terephthalate (Che *et al.*, 2006) that features a carboxylate-bridged chain motif. The title naphthalene-1,4-dicarboxylate adduct also adopts a layer motif. However, there is space between the chains that is large enough for a naphthalene-1,4-dicarboxylic acid molecule as well as a water molecule to fit in. Adjacent chains are linked by hydrogen bonds into a three-dimensional network.

### Experimental

Manganese dichloride dihydrate (0.02 g, 0.1 mmol), naphthalene-1,4-dicarboxylic acid (0.02 g, 0.1 mmol), 4-(1*H*-1,3,7,8-tetraazacyclopenta[*l*]phenanthren-2-yl)phenol4-(1*H*-imidazo[4,5-*f*][1,10]phenanthrolin-2-yl)phenol (0.03 g, 0.1 mmol) and water (15 ml) were heated in a 23 ml, Teflon-lined, stainless-steel Parr bomb at 408 K for 2 days. Crystals were obtained in 40% yield.

### Refinement

The naphthalene-1,4-dicarboxylic acid is disordered over a center-of-inversion. The fused-ring portion was refined as a rigid naphthalene group of 1.39 Å sides; the occupancy is 0.5. The C–O distances were restrained to 1.25±0.01 Å and the C_carboxyl_–C_aryl_ distances to 1.50±0.01 Å. Other restrained were applied to the carboxyl parts. The anisotropic displacement factors of the carbon and oxygen atoms were restrained to be nearly isotropic. The acid H atoms were arbitrarily placed on the carboxyl parts.

The carbon- and nitrogen-bound H atoms were placed in calculated positions [C–H 0.93, N–H 0.86, O–H 0.82 Å and *U*_iso_(H) 1.2*U*_eq_(C,*N*,*O*)], and were included in the refinement in the riding-model approximation. For one of the *N*-heterocycles, the amino –NH group is ordered whereas for the other, it is disordered. For the second *N*-heterocycle, hydrogen atoms of 0.5 occupancy were placed on the two nitrogen atoms. The water H-atoms were placed in chemically sensible positions on the basis of hydrogen bonds.

The final difference Fourier map had a large peak near H14, but this could not be modeled as a water molecule.

### Crystal data

**Table:**

[Mn(C_12_H_6_O_4_)(C_19_H_12_N_4_O)_2_]·0.5C_12_H_8_O_4_·H_2_O	*F*_000_ = 4200
*M**_r_* = 1019.87	*D*_x_ = 1.536 Mg m^−^^3^
Monoclinic, *C*2/*c*	Mo *K*α radiation λ = 0.71073 Å
Hall symbol: -C 2yc	Cell parameters from 24704 reflections
*a* = 48.398 (15) Å	θ = 3.0–27.5º
*b* = 9.089 (2) Å	µ = 0.38 mm^−^^1^
*c* = 20.598 (6) Å	*T* = 295 (2) K
β = 103.20 (1)º	Block, brown
*V* = 8821 (4) Å^3^	0.31 × 0.25 × 0.18 mm
*Z* = 8	

### Data collection

**Table:**

Rigaku R-AXIS RAPID diffractometer	10014 independent reflections
Radiation source: fine-focus sealed tube	5868 reflections with *I* > 2σ(*I*)
Monochromator: graphite	*R*_int_ = 0.084
Detector resolution: 10 pixels mm^-1^	θ_max_ = 27.5º
*T* = 295(2) K	θ_min_ = 3.0º
ω scans	*h* = −62→62
Absorption correction: multi-scan(ABSCOR; Higashi, 1995)	*k* = −11→11
*T*_min_ = 0.820, *T*_max_ = 1.000	*l* = −25→26
40785 measured reflections	

### Refinement

**Table:**

Refinement on *F*^2^	Secondary atom site location: difference Fourier map
Least-squares matrix: full	Hydrogen site location: inferred from neighbouring sites
*R*[*F*^2^ > 2σ(*F*^2^)] = 0.060	H-atom parameters constrained
*wR*(*F*^2^) = 0.170	*w* = 1/[σ^2^(*F*_o_^2^) + (0.0782*P*)^2^ + 7.7802*P*] where *P* = (*F*_o_^2^ + 2*F*_c_^2^)/3
*S* = 1.03	(Δ/σ)_max_ = 0.001
10014 reflections	Δρ_max_ = 1.35 e Å^−^^3^
717 parameters	Δρ_min_ = −0.42 e Å^−^^3^
116 restraints	Extinction correction: none
Primary atom site location: structure-invariant direct methods	

### Fractional atomic coordinates and isotropic or equivalent isotropic displacement parameters (Å^2^)

**Table:**

	*x*	*y*	*z*	*U*_iso_*/*U*_eq_	Occ. (<1)
Mn1	0.648562 (11)	0.41289 (6)	0.51035 (2)	0.03142 (15)	
O1	0.66266 (5)	0.5844 (3)	0.45399 (11)	0.0379 (6)	
O2	0.70748 (5)	0.5903 (3)	0.44417 (12)	0.0468 (7)	
O3	0.65765 (6)	0.4702 (3)	0.10116 (11)	0.0487 (7)	
O4	0.62358 (12)	0.3337 (5)	0.12178 (16)	0.137 (2)	
O5	0.80478 (5)	−0.5497 (3)	0.28409 (12)	0.0431 (6)	
H5O	0.7975	−0.5713	0.2453	0.052*	
O6	0.39480 (6)	0.0139 (4)	0.56796 (15)	0.0580 (8)	
H6O	0.3828	0.0538	0.5388	0.070*	
O1W	0.64402 (6)	0.8246 (3)	0.51341 (13)	0.0585 (8)	
H1W1	0.6381	0.7882	0.5440	0.070*	
H1W2	0.6500	0.7580	0.4935	0.070*	
N1	0.64987 (6)	0.2597 (3)	0.42332 (13)	0.0329 (6)	
N2	0.71343 (6)	−0.0949 (3)	0.35027 (13)	0.0326 (6)	
N3	0.74551 (6)	−0.0806 (3)	0.44653 (13)	0.0323 (6)	
H3N	0.7608	−0.0958	0.4765	0.039*	
N4	0.68967 (6)	0.2888 (3)	0.53848 (13)	0.0323 (6)	
N5	0.60125 (6)	0.4417 (3)	0.47014 (14)	0.0349 (7)	
N6	0.50889 (6)	0.2640 (4)	0.49973 (15)	0.0427 (8)	
H6N	0.4963	0.3127	0.4718	0.051*	0.50
N7	0.52850 (6)	0.1071 (3)	0.58045 (15)	0.0393 (7)	
H7N	0.5305	0.0422	0.6115	0.047*	0.50
N8	0.62546 (6)	0.2431 (3)	0.56234 (13)	0.0328 (6)	
C1	0.68180 (7)	0.5800 (4)	0.42007 (15)	0.0318 (7)	
C2	0.67164 (7)	0.5539 (4)	0.34578 (15)	0.0334 (8)	
C3	0.68747 (8)	0.4623 (4)	0.31553 (18)	0.0442 (9)	
H3	0.7046	0.4257	0.3404	0.053*	
C4	0.67855 (10)	0.4222 (5)	0.24807 (19)	0.0520 (11)	
H4	0.6904	0.3659	0.2282	0.062*	
C5	0.65281 (9)	0.4650 (4)	0.21179 (17)	0.0453 (10)	
C6	0.63544 (8)	0.5580 (4)	0.24055 (17)	0.0418 (9)	
C7	0.60821 (10)	0.6066 (6)	0.2051 (2)	0.0636 (14)	
H7	0.6006	0.5699	0.1626	0.076*	
C8	0.59313 (10)	0.7050 (7)	0.2321 (2)	0.0763 (17)	
H8	0.5750	0.7312	0.2087	0.092*	
C9	0.60437 (9)	0.7686 (6)	0.2949 (2)	0.0610 (13)	
H9	0.5944	0.8414	0.3115	0.073*	
C10	0.63004 (8)	0.7224 (4)	0.33118 (18)	0.0446 (9)	
H10	0.6375	0.7647	0.3726	0.053*	
C11	0.64569 (8)	0.6109 (4)	0.30714 (16)	0.0362 (8)	
C12	0.64374 (11)	0.4187 (5)	0.13943 (18)	0.0573 (12)	
C13	0.63068 (8)	0.2504 (4)	0.36630 (17)	0.0391 (8)	
H13	0.6143	0.3071	0.3612	0.047*	
C14	0.63352 (8)	0.1611 (4)	0.31385 (18)	0.0443 (9)	
H14	0.6195	0.1594	0.2745	0.053*	
C15	0.65707 (7)	0.0760 (4)	0.32064 (16)	0.0383 (8)	
H15	0.6592	0.0140	0.2862	0.046*	
C16	0.67804 (7)	0.0824 (4)	0.38000 (15)	0.0309 (7)	
C17	0.67358 (7)	0.1766 (4)	0.43056 (15)	0.0277 (7)	
C18	0.70380 (7)	−0.0002 (4)	0.39249 (15)	0.0302 (7)	
C19	0.73844 (7)	−0.1426 (4)	0.38480 (16)	0.0319 (7)	
C20	0.75594 (7)	−0.2469 (4)	0.35910 (15)	0.0310 (7)	
C21	0.74440 (8)	−0.3234 (4)	0.30051 (17)	0.0375 (8)	
H21	0.7256	−0.3066	0.2787	0.045*	
C22	0.76036 (7)	−0.4235 (4)	0.27433 (16)	0.0363 (8)	
H22	0.7523	−0.4730	0.2350	0.044*	
C23	0.78818 (7)	−0.4505 (4)	0.30626 (16)	0.0326 (8)	
C24	0.80001 (8)	−0.3755 (4)	0.36495 (17)	0.0394 (9)	
H24	0.8187	−0.3932	0.3868	0.047*	
C25	0.78397 (8)	−0.2747 (4)	0.39081 (17)	0.0388 (8)	
H25	0.7921	−0.2249	0.4300	0.047*	
C26	0.72353 (7)	0.0112 (4)	0.45189 (15)	0.0305 (7)	
C27	0.72016 (7)	0.1082 (4)	0.50390 (15)	0.0303 (7)	
C28	0.69495 (7)	0.1906 (4)	0.49268 (15)	0.0289 (7)	
C29	0.73988 (8)	0.1291 (4)	0.56409 (16)	0.0368 (8)	
H29	0.7567	0.0751	0.5734	0.044*	
C30	0.73462 (8)	0.2288 (4)	0.60954 (17)	0.0417 (9)	
H30	0.7478	0.2448	0.6494	0.050*	
C31	0.70901 (8)	0.3062 (4)	0.59476 (16)	0.0370 (8)	
H31	0.7054	0.3731	0.6260	0.044*	
C32	0.58966 (8)	0.5338 (4)	0.42155 (17)	0.0421 (9)	
H32	0.6017	0.5940	0.4039	0.051*	
C33	0.56062 (8)	0.5453 (5)	0.39549 (19)	0.0472 (10)	
H33	0.5536	0.6091	0.3603	0.057*	
C34	0.54270 (8)	0.4625 (4)	0.42190 (18)	0.0448 (9)	
H34	0.5232	0.4703	0.4059	0.054*	
C35	0.55415 (7)	0.3645 (4)	0.47394 (17)	0.0352 (8)	
C36	0.58368 (7)	0.3551 (4)	0.49587 (16)	0.0308 (7)	
C37	0.53799 (7)	0.2739 (4)	0.50782 (17)	0.0375 (8)	
C38	0.50402 (8)	0.1628 (4)	0.54421 (18)	0.0390 (8)	
C39	0.47567 (8)	0.1240 (4)	0.55059 (18)	0.0398 (9)	
C40	0.45204 (8)	0.1852 (5)	0.5075 (2)	0.0466 (9)	
H40	0.4546	0.2507	0.4746	0.056*	
C41	0.42516 (8)	0.1502 (4)	0.51280 (19)	0.0441 (9)	
H41	0.4096	0.1918	0.4835	0.053*	
C42	0.42095 (8)	0.0535 (4)	0.56145 (19)	0.0418 (9)	
C43	0.44413 (9)	−0.0074 (5)	0.6052 (2)	0.0502 (10)	
H43	0.4415	−0.0719	0.6383	0.060*	
C44	0.47111 (9)	0.0282 (5)	0.59918 (19)	0.0482 (10)	
H44	0.4866	−0.0133	0.6285	0.058*	
C45	0.54997 (7)	0.1768 (4)	0.55707 (16)	0.0360 (8)	
C46	0.58004 (7)	0.1575 (4)	0.57679 (16)	0.0317 (7)	
C47	0.59665 (7)	0.2488 (4)	0.54633 (15)	0.0301 (7)	
C48	0.59376 (8)	0.0503 (4)	0.62186 (18)	0.0423 (9)	
H48	0.5833	−0.0148	0.6417	0.051*	
C49	0.62277 (8)	0.0435 (4)	0.63611 (18)	0.0422 (9)	
H49	0.6324	−0.0275	0.6653	0.051*	
C50	0.63774 (8)	0.1443 (4)	0.60637 (17)	0.0391 (9)	
H50	0.6575	0.1416	0.6182	0.047*	
O7	0.57620 (11)	0.3027 (6)	0.1777 (3)	0.0515 (14)	0.50
H7O	0.5886	0.3112	0.1568	0.062*	0.50
O8	0.54490 (15)	0.4380 (7)	0.1031 (3)	0.0662 (18)	0.50
O9	0.47578 (12)	0.3147 (7)	0.3757 (3)	0.0619 (16)	0.50
O10	0.46652 (12)	0.0992 (7)	0.3243 (3)	0.0651 (17)	0.50
H10O	0.4575	0.0738	0.3517	0.078*	0.50
C51	0.55115 (11)	0.3657 (5)	0.1545 (2)	0.0409 (17)	0.50
C52	0.53036 (7)	0.3410 (4)	0.19789 (16)	0.0385 (17)	0.50
C53	0.52596 (9)	0.1961 (4)	0.2146 (2)	0.049 (2)	0.50
H53	0.5352	0.1200	0.1981	0.059*	0.50
C54	0.50771 (9)	0.1650 (3)	0.2560 (2)	0.047 (2)	0.50
H54	0.5048	0.0681	0.2672	0.056*	0.50
C55	0.49387 (8)	0.2788 (4)	0.28073 (17)	0.0461 (19)	0.50
C56	0.49827 (9)	0.4237 (4)	0.2640 (2)	0.042 (3)	0.50
C57	0.51652 (9)	0.4548 (3)	0.22257 (19)	0.041 (2)	0.50
C58	0.52092 (14)	0.5997 (4)	0.2058 (3)	0.044 (2)	0.50
H58	0.5331	0.6205	0.1781	0.053*	0.50
C59	0.50708 (19)	0.7134 (3)	0.2305 (4)	0.047 (3)	0.50
H59	0.5100	0.8104	0.2193	0.057*	0.50
C60	0.48883 (19)	0.6823 (4)	0.2719 (4)	0.068 (4)	0.50
H60	0.4796	0.7584	0.2884	0.082*	0.50
C61	0.48443 (14)	0.5374 (5)	0.2887 (3)	0.057 (3)	0.50
H61	0.4722	0.5166	0.3164	0.068*	0.50
C62	0.47719 (9)	0.2234 (7)	0.3285 (3)	0.050 (2)	0.50

### Atomic displacement parameters (Å^2^)

**Table:**

	*U*^11^	*U*^22^	*U*^33^	*U*^12^	*U*^13^	*U*^23^
Mn1	0.0325 (3)	0.0381 (3)	0.0258 (2)	0.0015 (2)	0.0110 (2)	−0.0006 (2)
O1	0.0453 (15)	0.0431 (14)	0.0294 (12)	0.0010 (12)	0.0171 (11)	0.0011 (11)
O2	0.0342 (15)	0.0710 (19)	0.0338 (13)	0.0000 (14)	0.0048 (11)	−0.0056 (13)
O3	0.0654 (19)	0.0591 (17)	0.0245 (12)	0.0084 (15)	0.0161 (12)	0.0071 (12)
O4	0.217 (5)	0.161 (4)	0.0387 (18)	−0.144 (4)	0.040 (2)	−0.035 (2)
O5	0.0398 (15)	0.0600 (17)	0.0289 (12)	0.0147 (13)	0.0063 (11)	−0.0057 (12)
O6	0.0341 (15)	0.080 (2)	0.0612 (18)	−0.0089 (15)	0.0143 (13)	0.0063 (17)
O1W	0.063 (2)	0.0632 (19)	0.0460 (16)	0.0125 (16)	0.0062 (14)	−0.0068 (14)
N1	0.0311 (15)	0.0399 (16)	0.0290 (14)	0.0017 (13)	0.0099 (12)	−0.0012 (13)
N2	0.0337 (15)	0.0388 (16)	0.0262 (13)	0.0026 (13)	0.0087 (12)	−0.0015 (13)
N3	0.0321 (15)	0.0394 (16)	0.0254 (13)	0.0046 (13)	0.0063 (11)	−0.0030 (13)
N4	0.0352 (16)	0.0368 (16)	0.0260 (13)	0.0003 (13)	0.0092 (12)	−0.0023 (12)
N5	0.0364 (16)	0.0382 (16)	0.0323 (14)	0.0028 (13)	0.0124 (13)	0.0040 (13)
N6	0.0330 (17)	0.0523 (19)	0.0438 (17)	−0.0035 (15)	0.0109 (14)	0.0065 (16)
N7	0.0322 (16)	0.0495 (18)	0.0385 (16)	−0.0004 (14)	0.0128 (13)	0.0016 (14)
N8	0.0298 (15)	0.0368 (15)	0.0325 (14)	0.0026 (13)	0.0084 (12)	0.0012 (13)
C1	0.0367 (19)	0.0358 (18)	0.0249 (15)	0.0006 (16)	0.0112 (14)	0.0000 (15)
C2	0.0381 (19)	0.0400 (19)	0.0236 (15)	−0.0060 (16)	0.0100 (14)	0.0004 (14)
C3	0.045 (2)	0.056 (2)	0.0338 (18)	0.0049 (19)	0.0142 (17)	0.0013 (18)
C4	0.071 (3)	0.056 (3)	0.0351 (19)	0.001 (2)	0.025 (2)	−0.0081 (19)
C5	0.066 (3)	0.047 (2)	0.0250 (17)	−0.019 (2)	0.0147 (18)	−0.0020 (17)
C6	0.046 (2)	0.054 (2)	0.0257 (16)	−0.0174 (19)	0.0090 (16)	0.0095 (16)
C7	0.053 (3)	0.105 (4)	0.0291 (19)	−0.024 (3)	0.0025 (19)	0.016 (2)
C8	0.040 (3)	0.137 (5)	0.052 (3)	0.002 (3)	0.010 (2)	0.044 (3)
C9	0.052 (3)	0.085 (3)	0.052 (2)	0.020 (2)	0.023 (2)	0.029 (2)
C10	0.046 (2)	0.053 (2)	0.0374 (19)	0.0059 (19)	0.0164 (17)	0.0095 (18)
C11	0.040 (2)	0.045 (2)	0.0260 (16)	−0.0073 (17)	0.0123 (15)	0.0060 (15)
C12	0.091 (4)	0.056 (3)	0.0261 (18)	−0.027 (3)	0.016 (2)	−0.0040 (18)
C13	0.0334 (19)	0.048 (2)	0.0349 (18)	0.0104 (17)	0.0046 (15)	−0.0018 (17)
C14	0.040 (2)	0.057 (2)	0.0319 (18)	0.0058 (19)	0.0005 (16)	−0.0063 (18)
C15	0.037 (2)	0.050 (2)	0.0259 (16)	0.0047 (17)	0.0036 (14)	−0.0065 (16)
C16	0.0302 (17)	0.0365 (18)	0.0267 (15)	0.0009 (15)	0.0079 (13)	0.0009 (15)
C17	0.0292 (17)	0.0319 (17)	0.0240 (15)	−0.0016 (14)	0.0101 (13)	0.0026 (14)
C18	0.0328 (18)	0.0345 (18)	0.0242 (15)	−0.0004 (15)	0.0081 (13)	−0.0003 (14)
C19	0.0349 (19)	0.0378 (18)	0.0245 (15)	−0.0003 (16)	0.0100 (14)	0.0001 (14)
C20	0.0345 (18)	0.0346 (18)	0.0254 (15)	−0.0001 (15)	0.0102 (14)	0.0000 (14)
C21	0.0333 (19)	0.046 (2)	0.0328 (17)	0.0057 (17)	0.0059 (15)	−0.0018 (16)
C22	0.038 (2)	0.044 (2)	0.0261 (16)	0.0038 (17)	0.0054 (14)	−0.0039 (15)
C23	0.0329 (18)	0.0381 (19)	0.0286 (16)	0.0056 (15)	0.0105 (14)	0.0018 (15)
C24	0.0311 (19)	0.052 (2)	0.0328 (18)	0.0045 (17)	0.0033 (15)	−0.0039 (17)
C25	0.037 (2)	0.048 (2)	0.0299 (17)	0.0024 (17)	0.0034 (15)	−0.0071 (16)
C26	0.0332 (18)	0.0330 (17)	0.0268 (15)	0.0008 (15)	0.0100 (14)	0.0010 (14)
C27	0.0302 (17)	0.0366 (18)	0.0268 (15)	−0.0007 (15)	0.0120 (13)	0.0013 (14)
C28	0.0318 (18)	0.0303 (17)	0.0262 (15)	−0.0040 (14)	0.0101 (13)	0.0001 (14)
C29	0.0351 (19)	0.046 (2)	0.0290 (16)	0.0058 (16)	0.0063 (15)	−0.0019 (16)
C30	0.040 (2)	0.054 (2)	0.0295 (17)	−0.0006 (19)	0.0056 (16)	−0.0060 (17)
C31	0.039 (2)	0.043 (2)	0.0289 (17)	−0.0010 (17)	0.0090 (15)	−0.0066 (16)
C32	0.042 (2)	0.049 (2)	0.0361 (18)	−0.0013 (18)	0.0110 (16)	0.0100 (18)
C33	0.044 (2)	0.054 (2)	0.041 (2)	0.0009 (19)	0.0049 (18)	0.0148 (19)
C34	0.037 (2)	0.055 (2)	0.039 (2)	0.0008 (19)	0.0008 (16)	0.0106 (19)
C35	0.0342 (19)	0.042 (2)	0.0307 (17)	−0.0010 (16)	0.0091 (15)	0.0025 (16)
C36	0.0323 (18)	0.0346 (18)	0.0274 (16)	−0.0006 (15)	0.0105 (14)	−0.0029 (14)
C37	0.0311 (19)	0.050 (2)	0.0309 (17)	−0.0002 (17)	0.0063 (15)	0.0032 (17)
C38	0.036 (2)	0.045 (2)	0.0395 (19)	−0.0020 (17)	0.0160 (16)	0.0007 (17)
C39	0.037 (2)	0.046 (2)	0.0386 (19)	−0.0034 (17)	0.0139 (16)	−0.0045 (17)
C40	0.043 (2)	0.052 (2)	0.048 (2)	−0.0024 (19)	0.0168 (18)	0.0041 (19)
C41	0.039 (2)	0.051 (2)	0.043 (2)	−0.0001 (18)	0.0112 (17)	−0.0025 (19)
C42	0.035 (2)	0.050 (2)	0.043 (2)	−0.0077 (17)	0.0151 (16)	−0.0104 (18)
C43	0.046 (2)	0.062 (3)	0.046 (2)	−0.007 (2)	0.0171 (19)	0.006 (2)
C44	0.041 (2)	0.060 (3)	0.043 (2)	0.001 (2)	0.0106 (17)	0.010 (2)
C45	0.035 (2)	0.044 (2)	0.0308 (17)	−0.0026 (17)	0.0115 (15)	0.0003 (16)
C46	0.0331 (19)	0.0352 (18)	0.0270 (16)	0.0029 (15)	0.0071 (14)	−0.0005 (15)
C47	0.0318 (18)	0.0328 (17)	0.0262 (15)	−0.0006 (15)	0.0076 (14)	−0.0032 (14)
C48	0.044 (2)	0.045 (2)	0.0374 (19)	0.0030 (18)	0.0094 (17)	0.0073 (17)
C49	0.045 (2)	0.043 (2)	0.0391 (19)	0.0104 (18)	0.0096 (17)	0.0120 (17)
C50	0.037 (2)	0.046 (2)	0.0351 (18)	0.0063 (17)	0.0103 (16)	0.0060 (17)
O7	0.048 (3)	0.060 (3)	0.053 (3)	−0.014 (3)	0.025 (3)	0.002 (3)
O8	0.092 (5)	0.072 (4)	0.044 (3)	0.014 (3)	0.033 (3)	0.016 (3)
O9	0.052 (3)	0.088 (4)	0.050 (3)	0.004 (3)	0.021 (3)	0.023 (3)
O10	0.057 (4)	0.074 (4)	0.068 (4)	−0.012 (3)	0.023 (3)	0.030 (3)
C51	0.044 (4)	0.043 (4)	0.039 (4)	−0.007 (3)	0.016 (3)	−0.009 (3)
C52	0.039 (4)	0.041 (4)	0.036 (3)	−0.005 (3)	0.010 (3)	−0.001 (3)
C53	0.072 (5)	0.042 (4)	0.033 (4)	−0.005 (4)	0.013 (4)	0.000 (4)
C54	0.064 (7)	0.039 (3)	0.033 (4)	−0.013 (3)	0.004 (4)	0.002 (4)
C55	0.039 (4)	0.054 (4)	0.044 (4)	0.002 (4)	0.006 (3)	0.022 (4)
C56	0.046 (4)	0.045 (4)	0.029 (6)	0.003 (5)	−0.008 (4)	0.010 (3)
C57	0.045 (5)	0.041 (5)	0.031 (4)	−0.002 (4)	−0.002 (4)	0.005 (4)
C58	0.041 (5)	0.047 (5)	0.045 (4)	−0.004 (4)	0.008 (4)	−0.015 (4)
C59	0.065 (6)	0.027 (4)	0.050 (6)	−0.002 (4)	0.014 (5)	−0.001 (4)
C60	0.062 (7)	0.075 (6)	0.072 (8)	0.011 (6)	0.023 (6)	−0.009 (6)
C61	0.036 (5)	0.080 (7)	0.057 (6)	0.003 (5)	0.018 (4)	0.012 (5)
C62	0.036 (4)	0.061 (5)	0.055 (5)	−0.003 (4)	0.011 (4)	0.014 (4)

### Geometric parameters (Å, °)

**Table:**

Mn1—O1	2.146 (2)	C21—H21	0.9300
Mn1—O3^i^	2.108 (2)	C22—C23	1.379 (5)
Mn1—N1	2.282 (3)	C22—H22	0.9300
Mn1—N4	2.245 (3)	C23—C24	1.392 (5)
Mn1—N5	2.265 (3)	C24—C25	1.384 (5)
Mn1—N8	2.307 (3)	C24—H24	0.9300
O1—C1	1.282 (4)	C25—H25	0.9300
O2—C1	1.232 (4)	C26—C27	1.425 (4)
O3—C12	1.239 (5)	C27—C29	1.394 (4)
O3—Mn1^ii^	2.108 (2)	C27—C28	1.405 (5)
O4—C12	1.232 (5)	C29—C30	1.368 (5)
O5—C23	1.354 (4)	C29—H29	0.9300
O5—H5O	0.8200	C30—C31	1.397 (5)
O6—C42	1.352 (4)	C30—H30	0.9300
O6—H6O	0.8200	C31—H31	0.9300
O1W—H1W1	0.8201	C32—C33	1.389 (5)
O1W—H1W2	0.8200	C32—H32	0.9300
N1—C13	1.323 (4)	C33—C34	1.352 (5)
N1—C17	1.353 (4)	C33—H33	0.9300
N2—C19	1.329 (4)	C34—C35	1.407 (5)
N2—C18	1.379 (4)	C34—H34	0.9300
N3—C19	1.361 (4)	C35—C36	1.400 (5)
N3—C26	1.376 (4)	C35—C37	1.423 (5)
N3—H3N	0.8600	C36—C47	1.453 (5)
N4—C31	1.323 (4)	C37—C45	1.369 (5)
N4—C28	1.364 (4)	C38—C39	1.452 (5)
N5—C32	1.327 (4)	C39—C44	1.382 (5)
N5—C36	1.352 (4)	C39—C40	1.394 (5)
N6—C38	1.356 (5)	C40—C41	1.368 (5)
N6—C37	1.383 (4)	C40—H40	0.9300
N6—H6N	0.8600	C41—C42	1.383 (5)
N7—C38	1.347 (5)	C41—H41	0.9300
N7—C45	1.393 (4)	C42—C43	1.384 (5)
N7—H7N	0.8600	C43—C44	1.378 (5)
N8—C50	1.318 (4)	C43—H43	0.9300
N8—C47	1.359 (4)	C44—H44	0.9300
C1—C2	1.515 (4)	C45—C46	1.430 (5)
C2—C3	1.374 (5)	C46—C47	1.400 (5)
C2—C11	1.422 (5)	C46—C48	1.403 (5)
C3—C4	1.406 (5)	C48—C49	1.369 (5)
C3—H3	0.9300	C48—H48	0.9300
C4—C5	1.355 (6)	C49—C50	1.394 (5)
C4—H4	0.9300	C49—H49	0.9300
C5—C6	1.414 (6)	C50—H50	0.9300
C5—C12	1.514 (5)	O7—C51	1.327 (6)
C6—C7	1.423 (6)	O7—H7O	0.8201
C6—C11	1.431 (5)	O8—C51	1.223 (6)
C7—C8	1.351 (7)	O9—C62	1.291 (7)
C7—H7	0.9300	O10—C62	1.236 (7)
C8—C9	1.409 (7)	O10—H10O	0.8198
C8—H8	0.9300	C51—C52	1.507 (5)
C9—C10	1.362 (5)	C52—C53	1.3900
C9—H9	0.9300	C52—C57	1.3900
C10—C11	1.420 (5)	C53—C54	1.3900
C10—H10	0.9300	C53—H53	0.9300
C13—C14	1.383 (5)	C54—C55	1.3900
C13—H13	0.9300	C54—H54	0.9300
C14—C15	1.358 (5)	C55—C56	1.3900
C14—H14	0.9300	C55—C62	1.496 (5)
C15—C16	1.401 (4)	C56—C57	1.3900
C15—H15	0.9300	C56—C61	1.3900
C16—C17	1.402 (4)	C57—C58	1.3900
C16—C18	1.427 (5)	C58—C59	1.3900
C17—C28	1.455 (4)	C58—H58	0.9300
C18—C26	1.373 (4)	C59—C60	1.3900
C19—C20	1.449 (5)	C59—H59	0.9300
C20—C25	1.388 (5)	C60—C61	1.3900
C20—C21	1.394 (5)	C60—H60	0.9300
C21—C22	1.381 (5)	C61—H61	0.9300
			
O1—Mn1—O3^i^	95.0 (1)	C24—C25—H25	119.5
O1—Mn1—N1	87.1 (1)	C20—C25—H25	119.5
O1—Mn1—N4	97.6 (1)	C18—C26—N3	106.0 (3)
O1—Mn1—N5	97.9 (1)	C18—C26—C27	122.7 (3)
O1—Mn1—N8	169.9 (1)	N3—C26—C27	131.2 (3)
O3^i^—Mn1—N1	165.2 (1)	C29—C27—C28	117.8 (3)
O3^i^—Mn1—N4	91.8 (1)	C29—C27—C26	125.4 (3)
O3^i^—Mn1—N5	104.8 (1)	C28—C27—C26	116.8 (3)
O3^i^—Mn1—N8	87.1 (1)	N4—C28—C27	121.8 (3)
N1—Mn1—N4	73.4 (1)	N4—C28—C17	117.2 (3)
N1—Mn1—N5	89.5 (1)	C27—C28—C17	120.9 (3)
N1—Mn1—N8	93.4 (1)	C30—C29—C27	120.1 (3)
N4—Mn1—N5	156.2 (1)	C30—C29—H29	119.9
N4—Mn1—N8	92.2 (1)	C27—C29—H29	119.9
N5—Mn1—N8	72.0 (1)	C29—C30—C31	118.6 (3)
C1—O1—Mn1	129.1 (2)	C29—C30—H30	120.7
C12—O3—Mn1^ii^	136.2 (3)	C31—C30—H30	120.7
C23—O5—H5O	109.5	N4—C31—C30	123.1 (3)
C42—O6—H6O	109.5	N4—C31—H31	118.5
H1W1—O1W—H1W2	108.2	C30—C31—H31	118.5
C13—N1—C17	117.9 (3)	N5—C32—C33	123.7 (4)
C13—N1—Mn1	126.6 (2)	N5—C32—H32	118.1
C17—N1—Mn1	115.4 (2)	C33—C32—H32	118.1
C19—N2—C18	105.3 (3)	C34—C33—C32	119.2 (4)
C19—N3—C26	107.0 (3)	C34—C33—H33	120.4
C19—N3—H3N	126.5	C32—C33—H33	120.4
C26—N3—H3N	126.5	C33—C34—C35	118.8 (4)
C31—N4—C28	118.5 (3)	C33—C34—H34	120.6
C31—N4—Mn1	125.0 (2)	C35—C34—H34	120.6
C28—N4—Mn1	116.4 (2)	C36—C35—C34	118.7 (3)
C32—N5—C36	117.9 (3)	C36—C35—C37	116.2 (3)
C32—N5—Mn1	124.5 (2)	C34—C35—C37	125.1 (3)
C36—N5—Mn1	117.6 (2)	N5—C36—C35	121.7 (3)
C38—N6—C37	107.0 (3)	N5—C36—C47	117.4 (3)
C38—N6—H6N	126.5	C35—C36—C47	120.9 (3)
C37—N6—H6N	126.5	C45—C37—N6	107.1 (3)
C38—N7—C45	105.6 (3)	C45—C37—C35	123.3 (3)
C38—N7—H7N	127.2	N6—C37—C35	129.6 (3)
C45—N7—H7N	127.2	N7—C38—N6	111.3 (3)
C50—N8—C47	118.0 (3)	N7—C38—C39	126.1 (3)
C50—N8—Mn1	125.8 (2)	N6—C38—C39	122.7 (3)
C47—N8—Mn1	116.2 (2)	C44—C39—C40	118.0 (4)
O2—C1—O1	124.5 (3)	C44—C39—C38	122.0 (3)
O2—C1—C2	118.9 (3)	C40—C39—C38	120.0 (3)
O1—C1—C2	116.6 (3)	C41—C40—C39	120.9 (4)
C3—C2—C11	118.7 (3)	C41—C40—H40	119.6
C3—C2—C1	117.8 (3)	C39—C40—H40	119.6
C11—C2—C1	123.4 (3)	C40—C41—C42	120.4 (4)
C2—C3—C4	121.9 (4)	C40—C41—H41	119.8
C2—C3—H3	119.1	C42—C41—H41	119.8
C4—C3—H3	119.1	O6—C42—C41	122.5 (4)
C5—C4—C3	120.4 (4)	O6—C42—C43	117.8 (4)
C5—C4—H4	119.8	C41—C42—C43	119.7 (4)
C3—C4—H4	119.8	C44—C43—C42	119.4 (4)
C4—C5—C6	120.0 (3)	C44—C43—H43	120.3
C4—C5—C12	119.1 (4)	C42—C43—H43	120.3
C6—C5—C12	120.8 (4)	C43—C44—C39	121.6 (4)
C5—C6—C7	122.7 (4)	C43—C44—H44	119.2
C5—C6—C11	119.5 (4)	C39—C44—H44	119.2
C7—C6—C11	117.7 (4)	C37—C45—N7	109.1 (3)
C8—C7—C6	121.3 (4)	C37—C45—C46	121.4 (3)
C8—C7—H7	119.4	N7—C45—C46	129.6 (3)
C6—C7—H7	119.4	C47—C46—C48	118.3 (3)
C7—C8—C9	121.2 (4)	C47—C46—C45	116.8 (3)
C7—C8—H8	119.4	C48—C46—C45	124.8 (3)
C9—C8—H8	119.4	N8—C47—C46	122.1 (3)
C10—C9—C8	119.3 (5)	N8—C47—C36	116.8 (3)
C10—C9—H9	120.3	C46—C47—C36	121.1 (3)
C8—C9—H9	120.3	C49—C48—C46	118.7 (4)
C9—C10—C11	121.4 (4)	C49—C48—H48	120.6
C9—C10—H10	119.3	C46—C48—H48	120.6
C11—C10—H10	119.3	C48—C49—C50	119.1 (3)
C10—C11—C2	122.5 (3)	C48—C49—H49	120.4
C10—C11—C6	118.6 (3)	C50—C49—H49	120.4
C2—C11—C6	118.9 (3)	N8—C50—C49	123.5 (4)
O4—C12—O3	123.9 (4)	N8—C50—H50	118.2
O4—C12—C5	119.8 (4)	C49—C50—H50	118.2
O3—C12—C5	116.3 (4)	C51—O7—H7O	119.4
N1—C13—C14	123.8 (3)	C62—O10—H10O	119.1
N1—C13—H13	118.1	O8—C51—O7	124.6 (6)
C14—C13—H13	118.1	O8—C51—C52	121.8 (5)
C15—C14—C13	119.0 (3)	O7—C51—C52	113.7 (4)
C15—C14—H14	120.5	C53—C52—C57	120.0
C13—C14—H14	120.5	C53—C52—C51	116.7 (3)
C14—C15—C16	119.3 (3)	C57—C52—C51	123.3 (3)
C14—C15—H15	120.3	C52—C53—C54	120.0
C16—C15—H15	120.3	C52—C53—H53	120.0
C17—C16—C15	118.0 (3)	C54—C53—H53	120.0
C17—C16—C18	117.6 (3)	C55—C54—C53	120.0
C15—C16—C18	124.4 (3)	C55—C54—H54	120.0
N1—C17—C16	122.0 (3)	C53—C54—H54	120.0
N1—C17—C28	117.6 (3)	C54—C55—C56	120.0
C16—C17—C28	120.5 (3)	C54—C55—C62	111.6 (3)
C26—C18—N2	110.0 (3)	C56—C55—C62	128.2 (3)
C26—C18—C16	121.5 (3)	C55—C56—C57	120.0
N2—C18—C16	128.5 (3)	C55—C56—C61	120.0
N2—C19—N3	111.6 (3)	C57—C56—C61	120.0
N2—C19—C20	123.7 (3)	C58—C57—C56	120.0
N3—C19—C20	124.7 (3)	C58—C57—C52	120.0
C25—C20—C21	118.2 (3)	C56—C57—C52	120.0
C25—C20—C19	122.5 (3)	C57—C58—C59	120.0
C21—C20—C19	119.3 (3)	C57—C58—H58	120.0
C22—C21—C20	121.2 (3)	C59—C58—H58	120.0
C22—C21—H21	119.4	C58—C59—C60	120.0
C20—C21—H21	119.4	C58—C59—H59	120.0
C23—C22—C21	120.2 (3)	C60—C59—H59	120.0
C23—C22—H22	119.9	C61—C60—C59	120.0
C21—C22—H22	119.9	C61—C60—H60	120.0
O5—C23—C22	123.2 (3)	C59—C60—H60	120.0
O5—C23—C24	117.4 (3)	C60—C61—C56	120.0
C22—C23—C24	119.4 (3)	C60—C61—H61	120.0
C25—C24—C23	120.2 (3)	C56—C61—H61	120.0
C25—C24—H24	119.9	O10—C62—O9	123.2 (6)
C23—C24—H24	119.9	O10—C62—C55	122.7 (6)
C24—C25—C20	120.9 (3)	O9—C62—C55	114.1 (5)
			
O3^i^—Mn1—O1—C1	122.4 (3)	C19—N3—C26—C18	0.4 (4)
N4—Mn1—O1—C1	30.0 (3)	C19—N3—C26—C27	−176.7 (3)
N5—Mn1—O1—C1	−131.9 (3)	C18—C26—C27—C29	−177.3 (3)
N1—Mn1—O1—C1	−42.8 (3)	N3—C26—C27—C29	−0.6 (6)
N8—Mn1—O1—C1	−136.0 (5)	C18—C26—C27—C28	1.8 (5)
O3^i^—Mn1—N1—C13	−177.0 (4)	N3—C26—C27—C28	178.4 (3)
O1—Mn1—N1—C13	−78.7 (3)	C31—N4—C28—C27	0.1 (5)
N4—Mn1—N1—C13	−177.5 (3)	Mn1—N4—C28—C27	178.8 (2)
N5—Mn1—N1—C13	19.3 (3)	C31—N4—C28—C17	−178.5 (3)
N8—Mn1—N1—C13	91.2 (3)	Mn1—N4—C28—C17	0.2 (4)
O3^i^—Mn1—N1—C17	−0.7 (6)	C29—C27—C28—N4	0.3 (5)
O1—Mn1—N1—C17	97.6 (2)	C26—C27—C28—N4	−178.8 (3)
N4—Mn1—N1—C17	−1.2 (2)	C29—C27—C28—C17	178.9 (3)
N5—Mn1—N1—C17	−164.4 (2)	C26—C27—C28—C17	−0.2 (4)
N8—Mn1—N1—C17	−92.5 (2)	N1—C17—C28—N4	−1.3 (4)
O3^i^—Mn1—N4—C31	−0.8 (3)	C16—C17—C28—N4	177.4 (3)
O1—Mn1—N4—C31	94.4 (3)	N1—C17—C28—C27	−179.9 (3)
N5—Mn1—N4—C31	−135.3 (3)	C16—C17—C28—C27	−1.3 (5)
N1—Mn1—N4—C31	179.1 (3)	C28—C27—C29—C30	−1.0 (5)
N8—Mn1—N4—C31	−88.0 (3)	C26—C27—C29—C30	178.0 (3)
O3^i^—Mn1—N4—C28	−179.4 (2)	C27—C29—C30—C31	1.2 (6)
O1—Mn1—N4—C28	−84.1 (2)	C28—N4—C31—C30	0.1 (5)
N5—Mn1—N4—C28	46.1 (4)	Mn1—N4—C31—C30	−178.5 (3)
N1—Mn1—N4—C28	0.5 (2)	C29—C30—C31—N4	−0.7 (6)
N8—Mn1—N4—C28	93.4 (2)	C36—N5—C32—C33	−0.5 (6)
O3^i^—Mn1—N5—C32	102.6 (3)	Mn1—N5—C32—C33	177.0 (3)
O1—Mn1—N5—C32	5.3 (3)	N5—C32—C33—C34	2.3 (6)
N4—Mn1—N5—C32	−124.9 (3)	C32—C33—C34—C35	−1.6 (6)
N1—Mn1—N5—C32	−81.7 (3)	C33—C34—C35—C36	−0.8 (6)
N8—Mn1—N5—C32	−175.4 (3)	C33—C34—C35—C37	178.1 (4)
O3^i^—Mn1—N5—C36	−79.9 (3)	C32—N5—C36—C35	−2.0 (5)
O1—Mn1—N5—C36	−177.2 (2)	Mn1—N5—C36—C35	−179.7 (3)
N4—Mn1—N5—C36	52.6 (4)	C32—N5—C36—C47	176.5 (3)
N1—Mn1—N5—C36	95.8 (2)	Mn1—N5—C36—C47	−1.1 (4)
N8—Mn1—N5—C36	2.0 (2)	C34—C35—C36—N5	2.7 (5)
O3^i^—Mn1—N8—C50	−75.1 (3)	C37—C35—C36—N5	−176.3 (3)
O1—Mn1—N8—C50	−177.4 (5)	C34—C35—C36—C47	−175.9 (3)
N4—Mn1—N8—C50	16.5 (3)	C37—C35—C36—C47	5.2 (5)
N5—Mn1—N8—C50	178.3 (3)	C38—N6—C37—C45	0.4 (4)
N1—Mn1—N8—C50	90.0 (3)	C38—N6—C37—C35	−179.4 (4)
O3^i^—Mn1—N8—C47	103.7 (2)	C36—C35—C37—C45	−3.0 (5)
O1—Mn1—N8—C47	1.5 (7)	C34—C35—C37—C45	178.1 (4)
N4—Mn1—N8—C47	−164.6 (2)	C36—C35—C37—N6	176.8 (4)
N5—Mn1—N8—C47	−2.8 (2)	C34—C35—C37—N6	−2.1 (7)
N1—Mn1—N8—C47	−91.1 (2)	C45—N7—C38—N6	−0.3 (4)
Mn1—O1—C1—O2	−80.7 (4)	C45—N7—C38—C39	−179.8 (4)
Mn1—O1—C1—C2	96.7 (3)	C37—N6—C38—N7	−0.1 (4)
O2—C1—C2—C3	37.7 (5)	C37—N6—C38—C39	179.4 (3)
O1—C1—C2—C3	−139.9 (4)	N7—C38—C39—C44	3.9 (6)
O2—C1—C2—C11	−146.9 (4)	N6—C38—C39—C44	−175.5 (4)
O1—C1—C2—C11	35.6 (5)	N7—C38—C39—C40	−176.9 (4)
C11—C2—C3—C4	−0.8 (6)	N6—C38—C39—C40	3.7 (6)
C1—C2—C3—C4	174.8 (3)	C44—C39—C40—C41	−0.6 (6)
C2—C3—C4—C5	−4.7 (6)	C38—C39—C40—C41	−179.8 (4)
C3—C4—C5—C6	3.8 (6)	C39—C40—C41—C42	0.2 (6)
C3—C4—C5—C12	−179.4 (4)	C40—C41—C42—O6	−178.9 (4)
C4—C5—C6—C7	−179.9 (4)	C40—C41—C42—C43	0.4 (6)
C12—C5—C6—C7	3.4 (6)	O6—C42—C43—C44	178.7 (4)
C4—C5—C6—C11	2.4 (6)	C41—C42—C43—C44	−0.6 (6)
C12—C5—C6—C11	−174.3 (3)	C42—C43—C44—C39	0.2 (7)
C5—C6—C7—C8	−174.5 (4)	C40—C39—C44—C43	0.3 (6)
C11—C6—C7—C8	3.3 (6)	C38—C39—C44—C43	179.6 (4)
C6—C7—C8—C9	3.1 (7)	N6—C37—C45—N7	−0.7 (4)
C7—C8—C9—C10	−4.7 (7)	C35—C37—C45—N7	179.2 (3)
C8—C9—C10—C11	−0.3 (6)	N6—C37—C45—C46	178.6 (3)
C9—C10—C11—C2	−175.8 (4)	C35—C37—C45—C46	−1.6 (6)
C9—C10—C11—C6	6.6 (5)	C38—N7—C45—C37	0.6 (4)
C3—C2—C11—C10	−170.6 (3)	C38—N7—C45—C46	−178.5 (4)
C1—C2—C11—C10	14.0 (5)	C37—C45—C46—C47	3.9 (5)
C3—C2—C11—C6	6.9 (5)	N7—C45—C46—C47	−177.1 (3)
C1—C2—C11—C6	−168.4 (3)	C37—C45—C46—C48	−173.3 (3)
C5—C6—C11—C10	169.9 (3)	N7—C45—C46—C48	5.8 (6)
C7—C6—C11—C10	−8.0 (5)	C50—N8—C47—C46	2.4 (5)
C5—C6—C11—C2	−7.8 (5)	Mn1—N8—C47—C46	−176.6 (2)
C7—C6—C11—C2	174.4 (3)	C50—N8—C47—C36	−177.8 (3)
Mn1^ii^—O3—C12—O4	53.9 (8)	Mn1—N8—C47—C36	3.3 (4)
Mn1^ii^—O3—C12—C5	−126.4 (4)	C48—C46—C47—N8	−4.4 (5)
C4—C5—C12—O4	113.9 (6)	C45—C46—C47—N8	178.2 (3)
C6—C5—C12—O4	−69.4 (7)	C48—C46—C47—C36	175.8 (3)
C4—C5—C12—O3	−65.9 (6)	C45—C46—C47—C36	−1.6 (5)
C6—C5—C12—O3	110.9 (5)	N5—C36—C47—N8	−1.5 (4)
C17—N1—C13—C14	−0.2 (6)	C35—C36—C47—N8	177.1 (3)
Mn1—N1—C13—C14	176.1 (3)	N5—C36—C47—C46	178.4 (3)
N1—C13—C14—C15	0.8 (6)	C35—C36—C47—C46	−3.0 (5)
C13—C14—C15—C16	−1.0 (6)	C47—C46—C48—C49	2.6 (5)
C14—C15—C16—C17	0.6 (5)	C45—C46—C48—C49	179.7 (4)
C14—C15—C16—C18	−179.1 (3)	C46—C48—C49—C50	1.0 (6)
C13—N1—C17—C16	−0.3 (5)	C47—N8—C50—C49	1.5 (5)
Mn1—N1—C17—C16	−176.9 (2)	Mn1—N8—C50—C49	−179.7 (3)
C13—N1—C17—C28	178.3 (3)	C48—C49—C50—N8	−3.2 (6)
Mn1—N1—C17—C28	1.7 (4)	O8—C51—C52—C53	127.7 (3)
C15—C16—C17—N1	0.1 (5)	O7—C51—C52—C53	−52.4 (3)
C18—C16—C17—N1	179.8 (3)	O8—C51—C52—C57	−54.2 (3)
C15—C16—C17—C28	−178.5 (3)	O7—C51—C52—C57	125.7 (3)
C18—C16—C17—C28	1.2 (5)	C57—C52—C53—C54	0.0
C19—N2—C18—C26	1.5 (4)	C51—C52—C53—C54	178.2 (2)
C19—N2—C18—C16	179.4 (3)	C52—C53—C54—C55	0.0
C17—C16—C18—C26	0.3 (5)	C53—C54—C55—C56	0.0
C15—C16—C18—C26	180.0 (3)	C53—C54—C55—C62	−175.3 (2)
C17—C16—C18—N2	−177.4 (3)	C54—C55—C56—C57	0.0
C15—C16—C18—N2	2.3 (6)	C62—C55—C56—C57	174.4 (2)
C18—N2—C19—N3	−1.2 (4)	C54—C55—C56—C61	180.0
C18—N2—C19—C20	179.2 (3)	C62—C55—C56—C61	−5.6 (2)
C26—N3—C19—N2	0.6 (4)	C55—C56—C57—C58	180.0
C26—N3—C19—C20	−179.9 (3)	C61—C56—C57—C58	0.0
N2—C19—C20—C25	168.0 (3)	C55—C56—C57—C52	0.0
N3—C19—C20—C25	−11.5 (5)	C61—C56—C57—C52	180.0
N2—C19—C20—C21	−12.3 (5)	C53—C52—C57—C58	180.0
N3—C19—C20—C21	168.2 (3)	C51—C52—C57—C58	2.0 (2)
C25—C20—C21—C22	−0.4 (5)	C53—C52—C57—C56	0.0
C19—C20—C21—C22	179.9 (3)	C51—C52—C57—C56	−178.0 (2)
C20—C21—C22—C23	0.4 (6)	C56—C57—C58—C59	0.0
C21—C22—C23—O5	178.4 (3)	C52—C57—C58—C59	180.0
C21—C22—C23—C24	−0.2 (5)	C57—C58—C59—C60	0.0
O5—C23—C24—C25	−178.8 (3)	C58—C59—C60—C61	0.0
C22—C23—C24—C25	−0.2 (6)	C59—C60—C61—C56	0.0
C23—C24—C25—C20	0.2 (6)	C55—C56—C61—C60	180.0
C21—C20—C25—C24	0.0 (5)	C57—C56—C61—C60	0.0
C19—C20—C25—C24	179.8 (3)	C54—C55—C62—O10	−30.3 (3)
N2—C18—C26—N3	−1.1 (4)	C56—C55—C62—O10	154.9 (3)
C16—C18—C26—N3	−179.2 (3)	C54—C55—C62—O9	147.5 (3)
N2—C18—C26—C27	176.2 (3)	C56—C55—C62—O9	−27.3 (3)
C16—C18—C26—C27	−1.8 (5)		

Symmetry codes: (i) *x*, −*y*+1, *z*+1/2; (ii) *x*, −*y*+1, *z*−1/2.

### Hydrogen-bond geometry (Å, °)

**Table:**

*D*—H···*A*	*D*—H	H···*A*	*D*···*A*	*D*—H···*A*
O5—H5O···N2^iii^	0.82	1.93	2.737 (4)	168
O6—H6O···O1W^iv^	0.82	1.85	2.656 (4)	168
N3—H3N···O2^v^	0.86	1.97	2.813 (4)	166
N6—H6N···O9	0.86	2.00	2.728 (6)	142
N7—H7N···O10^vi^	0.86	1.83	2.685 (6)	178
O1W—H1W2···O1	0.82	1.94	2.754 (4)	173
O1W—H1W1···O4^i^	0.82	2.19	3.007 (6)	173

Symmetry codes: (iii) −*x*+3/2, *y*−1/2, −*z*+1/2; (iv) −*x*+1, −*y*+1, −*z*+1; (v) −*x*+3/2, −*y*+1/2, −*z*+1; (vi) −*x*+1, −*y*, −*z*+1; (i) *x*, −*y*+1, *z*+1/2.

## References

[bb1] Barbour, L. J. (2001). *J. Supramol. Chem.* **1**, 189–191.

[bb2] Che, G.-B., Lin, X.-F. & Liu, C.-B. (2006). *Acta Cryst.* E**62**, m1456–m1458.

[bb3] Dolomanov, O. V., Blake, A. J., Champness, N. R. & Schröder, M. (2003). *J. Appl. Cryst.* **36**, 1283–1284.

[bb4] Higashi, T. (1995). *ABSCOR* Rigaku Corporation, Tokyo, Japan.

[bb5] Li, H.-D., Liu, Y., Xu, M.-L. & Ng, S. W. (2008). *Acta Cryst.* E**64**, m704–m705. 10.1107/S1600536808010787 PMC2961267 21202236

[bb6] Rigaku (1998). *RAPID-AUTO* Rigaku Corporation, Tokyo, Japan.

[bb7] Rigaku/MSC (2002). *CrystalStructure* Rigaku/MSC, The Woodlands, Texas, USA.

[bb8] Sheldrick, G. M. (2008). *Acta Cryst.* A**64**, 112–122. 10.1107/S0108767307043930 18156677

[bb9] Westrip, S. P. (2008). *publCIF* In preparation.

